# Structural Analysis of the Regulatory Domain of ExsA, a Key Transcriptional Regulator of the Type Three Secretion System in *Pseudomonas aeruginosa*

**DOI:** 10.1371/journal.pone.0136533

**Published:** 2015-08-28

**Authors:** Manisha Shrestha, Yi Xiao, Howard Robinson, Florian D. Schubot

**Affiliations:** 1 Department of Biological Sciences, Virginia Polytechnic Institute & State University, Washington Street, Blacksburg, VA 24060, United States of America; 2 Biology Department, Brookhaven National Laboratory, Upton, NY 11973–5000, United States of America; Saint Louis University, UNITED STATES

## Abstract

*Pseudomonas aeruginosa* employs a type three secretion system to facilitate infections in mammalian hosts. The operons encoding genes of structural components of the secretion machinery and associated virulence factors are all under the control of the AraC-type transcriptional activator protein, ExsA. ExsA belongs to a unique subfamily of AraC-proteins that is regulated through protein-protein contacts rather than small molecule ligands. Prior to infection, ExsA is inhibited through a direct interaction with the anti-activator ExsD. To activate ExsA upon host cell contact this interaction is disrupted by the anti-antiactivator protein ExsC. Here we report the crystal structure of the regulatory domain of ExsA, which is known to mediate ExsA dimerization as well as ExsD binding. The crystal structure suggests two models for the ExsA dimer. Both models confirmed the previously shown involvement of helix α-3 in ExsA dimerization but one also suggest a role for helix α-2. These structural data are supported by the observation that a mutation in α-2 greatly diminished the ability of ExsA to activate transcription *in vitro*. Additional *in vitro* transcription studies revealed that a conserved pocket, used by AraC and the related ToxT protein for the binding of small molecule regulators, although present in ExsA is not involved in binding of ExsD.

## Introduction

Gram-negative bacteria survive under a broad range of environmental conditions. Numerous species entertain mutualistic relationships or infects plant and animal hosts using a diverse array of virulence mechanisms. Perhaps the most prominent among these virulence mechanisms is the type three secretion system (T3SS). The T3SS consists of a needle apparatus, a varying array of exported toxins (or effectors), and, to ensure precisely timed expression, a specialized set of regulatory proteins [1–3]. The needle complex, broadly conserved across bacterial species, is used to transport toxins directly from the bacterial cytosol into the host cell cytoplasm. The types of the exported toxins differ among bacterial species as they appear tailored for specific hosts or niches within a host [4, 5]. While the molecular targets may vary, the secreted virulence factors generally fall into three functional categories: factors that act to subvert the host immune system [5–12], those that induce apoptosis [5, 11, 13–20], or, in case of intracellular bacteria, those that mediate engulfment by the host cell [4, 21–25]. Expression of T3SS-associated genes is usually timed to coincide with host infection. Host sensing is accomplished through a variety of mechanisms such as a shift in nutrient conditions, changes in temperature and physical contact with a host cell [3, 26–30].

A number of well-known mammalian pathogens are among the Gram-negative bacteria that employ T3SSs to facilitate infection. These include *Yersinia pestis*, *Salmonella enterica*, *Chlamydia species*, *Vibrio species*, and *Pseudomonas aeruginosa* [1, 2, 5, 31]. *P*.*aeruginosa* causes opportunistic acute and chronic infections in wide range of animal and plant hosts [10, 32–34]. The T3SS of *P*.*aruginosa*, transporting at least four potent cytotoxins into host cells, is essential for the establishment of acute infections [7, 14, 18–20, 35–40]; and there is some evidence to suggest that the T3SS is also important during the early stages of chronic infections [41–45]. Underlying the remarkable adaptability of *P*.*aeruginosa* to exploit a broad range of hosts are intricate regulatory networks formed by the biggest set of regulatory proteins among all known bacterial species. Presumably to preserve energy and avoid premature detection by the host organism expression of the T3SS is also coordinated by a complex network of signaling pathways [7, 29, 46–48]. One of these pathways, the ExsA-ExsC-ExsD-ExsE cascade, provides a direct link between bacterial host-cell contact and an upregulation of T3SS-related gene expression [49, 50]. Following a non-canonical mechanism, signaling is mediated by the formation and dissociation of three mutually exclusive protein-protein complexes [49–51]. Under non-inducing conditions the transcriptional activator ExsA is sequestered by the anti-activator protein ExsD [52]. Under these conditions the type three secretion chaperone ExsC and the 81 amino acid ExsE also form a tight complex [49–51]. Host-cell contact triggers opening of the basally expressed secretion apparatuses leading to ExsE export [49, 50]. The liberated ExsC binds ExsD to cause the release of ExsA [53]. The transcription factor ExsA in turn recruit RNA polymerase to the transcription initiation sites of the eleven promoters that control the expression of the T3SS genes [49, 50, 52, 54–57]. Over the years the details of the mechanism of transcription activation have been worked out and we have gained a fairly clear picture for the structural basis of the ExsC-ExsE and ExsC-ExsD interactions [56, 58]. In addition, the features of ExsA-dependent promoters have also been elucidated [59]. These promoters contain consensus regions similar to -35 and -10 sites of constitutive σ70-dependent promoters. However, while canonical -35 and -10 sites are usually spaced by 17 nucleotides the separation is 21 to 22 bases pairs for ExsA-dependent promoters, a spacing that was shown to be critical for ExsA-dependent transcription [59]. Each promoter contains two ExsA binding sites. One site overlaps the -35 element, the site also involved in Sigma-70 binding, while the second site encompasses the conserved adenine-rich region centered near the -51 position [60–63].

The ExsA protein consists of a ~100 amino acid carboxy-terminal domain (ExsA-CTD) and a ~170 amino acid amino-terminal domain (ExsA-NTD). The two domains are connected by a flexible linker. ExsA-CTD contains two helix-turn-helix motifs required for binding to T3SS associated promoters [64]. The ExsA-NTD mediates homo-dimerization but is also target for ExsD binding [54, 55]. Most recently, dimerization of the amino-terminal domain of ExsA was shown to be essential for not only stabilizing ExsA-DNA interactions but also for facilitating a structural change in ExsA that permits sequential binding of two ExsA molecules to the promoter [65]. ExsD inhibits ExsA function by interfering with both dimerization and promoter binding of ExsA [54, 55].

ExsA constitutes a particularly attractive target for the development of novel therapeutics because it belongs to the AraC family, which is comprised entirely of bacterial and fungal proteins but not represented in higher eukaryotes [66]. From a standpoint of drug design perhaps the most interesting targets in this signaling cascade are the ExsA-DNA and the ExsA-ExsD interfaces. Previous mutagenesis, functional, and interface mapping studies have provided a reasonably clear view of the ExsA-DNA interactions [60–62]. However, the interface of the ExsA-ExsD complex has so far resisted detailed analysis. AraC-type proteins such as ExsA are usually regulated through small-molecule ligands [66–70]. In the cases of ToxT and AraC these ligands that bind in a conserved pocket within the beta-barrel structure of the regulatory domain [66–70]. Here for the first time we report the structure for the regulatory domain of ExsA that belongs to a group of AraC-type transcriptional factors, which is regulated not by a small molecule ligand but by another protein [22, 23, 25, 71, 72]. Additional mutational analysis coupled with functional assays demonstrate that the conserved cavity in ExsA, although present, is not required for binding of ExsD *in vitro*, suggesting that this subfamily of AraC-proteins is regulated through interactions that involve distinct molecular interfaces.

## Results

### Crystal Structure of the regulatory domain of ExsA

**Fig 1:**
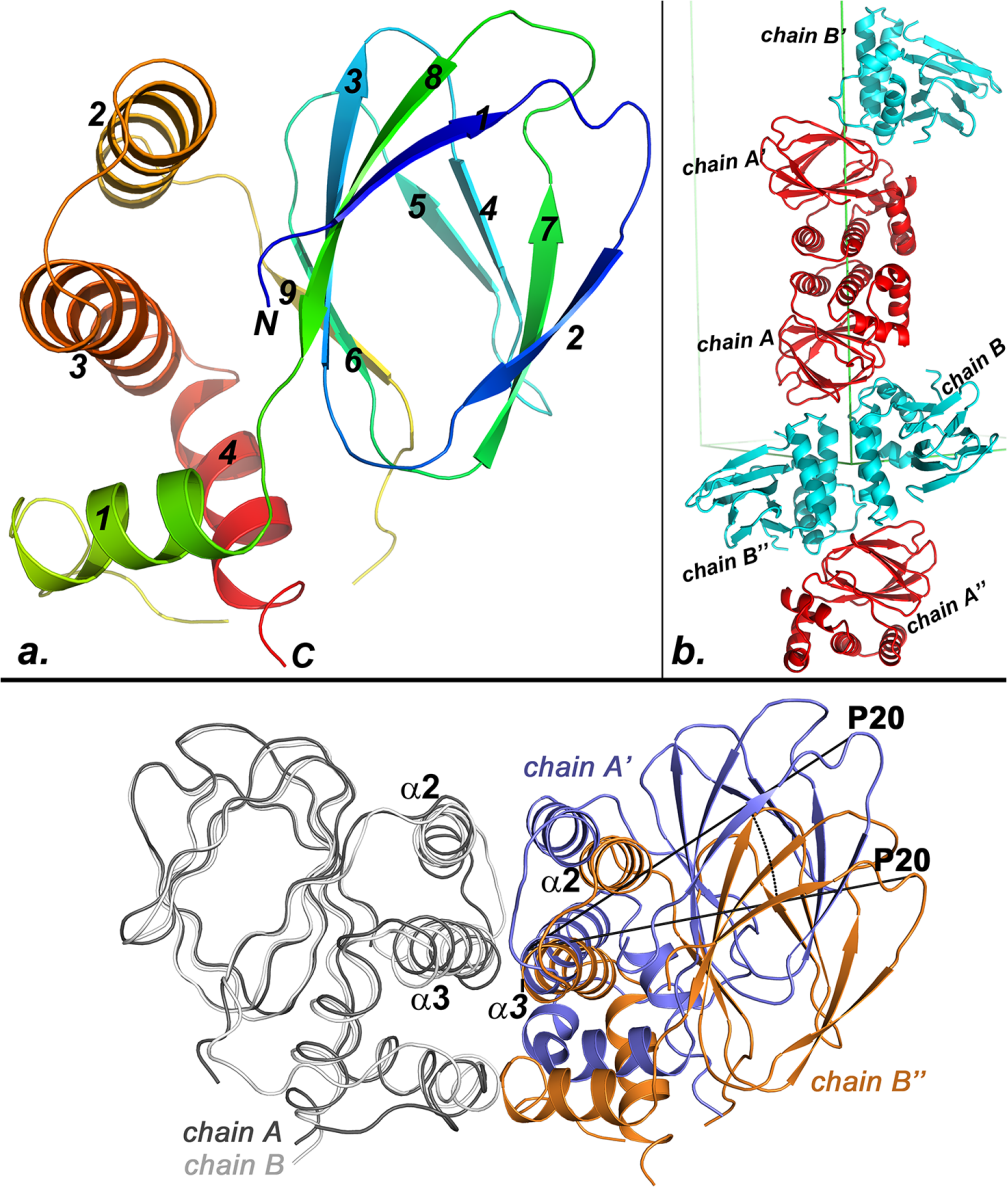
Crystal structure of the ExsA-NTD. **(A)** Model of a monomer encompassing amino acids 2–166 which produced clearly defined electron density. Blue to red rainbow coloring traces the backbone from the N to the C-terminus. Secondary structure elements are numbered. **(B)** Packing contacts in the crystal suggest the possible structure of the biological dimer. Chains A and B constitute the asymmetric unit of the crystal. Application of two crystallographic two-fold axes produces two additional pairs of chains labeled with a prime and a double-prime, respectively. Contacts between either chains A and A’ or between chains B and B” are proposed to mediate ExsA dimerization *in vivo*. (C) Shown in gray are the overlaid backbones traces of chains A and B. Also displayed are the symmetry-related molecules A’ and B” to highlight similarities and differences between the two possible quaternary structures. The B” molecule is rotated by approximately 23° around helix α-3. The rotation is visualized by marking the angle between the P20 residues of A’ and B” in the figure.

Because full-length ExsA resisted crystallization the protein was subjected to limited proteolysis with thermolysin. Remarkably, the digest produced large amounts of a single fragment that could be readily purified via gel filtration chromatography. LC-MS analysis revealed that this proteolytic product encompasses the entire ExsA-NTD (amino acids 2–178). Initial crystallization conditions were identified from commercial sparse matrix screening kits. Careful optimization yielded crystals that diffracted X-rays to about 2.5 Å resolution. Seleno-methionine-substituted sample was produced to resolve the phase problem [73], crystallized, and subjected to diffraction analysis at the absorption peak wavelength for Selenium. Single anomalous scattering phasing of these data with PHENIX [74] produced a readily traceable electron density map. Two molecules with very similar overall structures form the asymmetric unit of the crystal. The r.m.s.d. for the superposition of backbone atoms is about 2Å for the two chains. In chain A both termini encompassing residues 2 to 10 and 166–178, respectively produced no interpretable electron density. In chain B the termini were also flexible producing no density for residue ranges 2 to 9 and 167 to 178. A cartoon drawing of chain A is presented in Fig 1A. Despite low sequence similarity the overall fold of ExsA-NTD closely resembles those of the regulatory domains of AraC and ToxT. A structural alignment of ExsA-NTD with AraC-NTD produced an r.m.s.d. of 3.1Å for the backbone superposition with 16% sequence identity. The overlay of ExsA-NTD with ToxT-NTD gave an r.m.s.d. of 3.2Å for the backbone superposition but only 6% of the structurally aligned residues are identical.

### Helices α-2 and α-3 together form the core of the ExsA dimer interface

**Fig 2:**
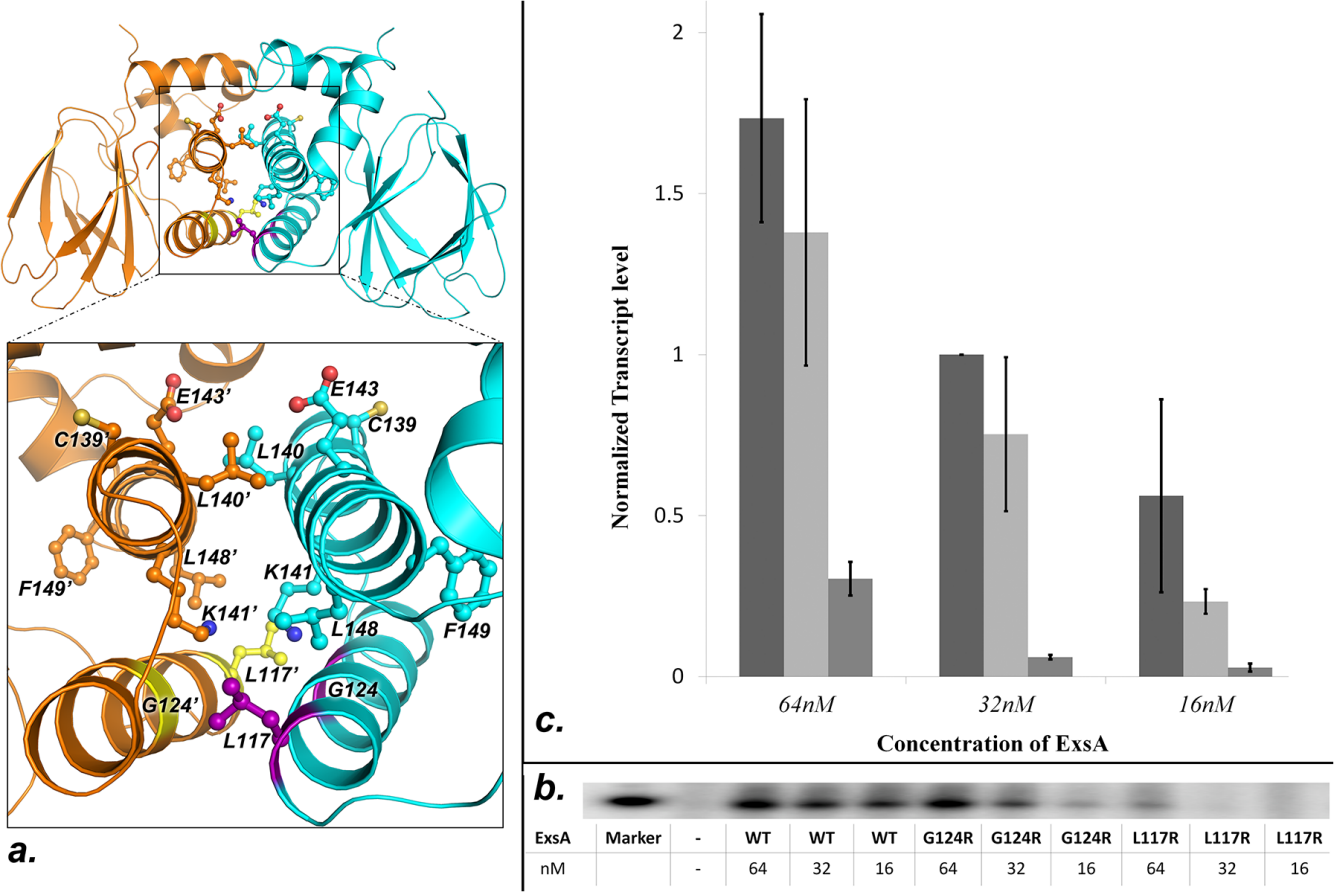
Mapping of the ExsA dimer interface. (A) The shown A/A’ ExsA-NTD interface suggests involvement of helix α-2 in ExsA dimerization. Previously identified interface residues are indicated in the same color as the protein backbone. G124 and L117 are colored violet and yellow in the respective molecules. (B) Shown is a sample gel of measurements testing the impact of the L117R and G124R mutations on the ability of ExsA to activate transcription *in vitro*. Three concentrations of each protein were tested to ensure that the experiments were conducted in a sensitive range. (C) Graphical representation of the *in vitro* transcription assays from triplicate experiments. Going from left to right: wtExsA, ExsAG124R, and ExsAL117R.

Overall, the fold of this domain is characterized by the formation of a beta barrel structure, which, in case of AraC and ToxT, houses the ligand binding pocket. The beta structure is flanked by two parallel carboxy-terminal helices α-2 and α-3 (Fig 1A). In AraC residues within these helices are critical for homo-dimerization by forming an antiparallel four-helix bundle wherein the two α-2 helices and the two α-3 helices are paired with each other [75]. More recently a mutational analysis of the corresponding region in ExsA demonstrated that α-3 residues C139, L140, K141, E143, L148, and F149 are important for optimal ExsA function [65]. The two molecules in the asymmetric unit are not interacting with each other through α-3. However, a dimer consistent with the experimental findings may be obtained from the ExsA-NTD crystal structure by generating crystallographic symmetry mates for the two independent molecules (Fig 1B). Our structural data suggest that L140, K141, E143, and L148, previously identified as critical for ExsA function [65], are all directly involved in ExsA dimerization (Fig 2A). The two resulting symmetric dimers consisting of either two chain A molecules (designated as A and A’ in Fig 1B) or two chain B molecules (designated as B and B” in Fig 1B) are not structurally identical but similar. Both provide a structural support for the previously published experimental findings that L140, K141, E143, and L148, which are all positioned on helix α-3 are directly involved in ExsA dimerization [65]. C139 and F149, on the other hand, are not directly positioned at the observed interface suggesting that mutating these residues affects ExsA dimerization indirectly (Fig 2A).

In Fig 1C chain A and chain B have been superposed and to observe the relative positioning of the respective symmetry mates. Compared to A’ in the A/A’ dimer the B” molecule is rotated by roughly 23° around its α-3 helix in the B/B’ dimer. The result of this rotation is a smaller number of interactions in the B/B” dimer. In the A/A’ dimer helices α-2 and α-3 form the core of the interface, whereas no contacts are observed between residues from helix α-2 in the B/B” dimer. A schematic representation of the non-bonding contacts in the two dimers is provided in S2 Fig. It is possible that packing pressures led to the differences between the two dimer interfaces observed in the crystal. Therefore, we set out to determine whether or not contacts mediated by α-2 as observed in the A/A’ dimer are biologically important. To examine this possibility we generated two additional ExsA variants based on our structural data. Because the structural model shows a large dimerization interface, we opted to introduce changes, G124 to arginine and L117 to arginine that should not only reduce the number of intermolecular contacts but also should actively disrupt dimerization (Fig 2A). Dimerization of ExsA is mediated by the ExsA-NTD. While this has not been explicitly shown for ExsA, the AraC-domains of other family members have been shown to facilitate DNA binding and recruitment of the RNA polymerase holoenzyme to their cognate promoters [76–78]. Therefore, a loss of transcription activation as the result of a point mutation in ExsA-NTD would provide indirect support for the hypothesized location of the dimer interface. Both, ExsA-G124R and ExsA-L117R expressed stably and could be readily purified (S2 Fig). In order to ensure that the activity measurements occurred within the sensitive range *in vitro* transcription assays were performed for three different concentrations of each of the ExsA variants. Although, ExsA-G124R appeared to trend toward inducing lower levels of transcription these differences were not statistically significant given the observed experimental error (P-values ranged from 0.3 to 0.19 for the three different concentrations (Fig 2B). The ExsA-L117R variant, on the other hand, was at least ten-fold attenuated compared to wild-type ExsA. Statistical analysis gave P values of less than 0.02 for the experiments conducted with 64 nM and 32 nM of ExsA-L117R. At 16 nM ExsA-L117R the lower transcript levels produced a larger experimental error so that the difference between the reactions conducted in the presence of wtExsA and those conducted with the ExsA-L117R variant was no longer statistically significant (P = 0.09). While the large experimental error does not allow us to conclude that G124 is positioned at the dimerization interface the data collectively support the hypothesis that, in addition to α-3, helix α-2 also has a functional role in ExsA dimerization as predicted by the structure of the A/A’ dimer.

### The cavity within the ExsA-NTD beta-barrel is not involved in ExsD binding

**Fig 3:**
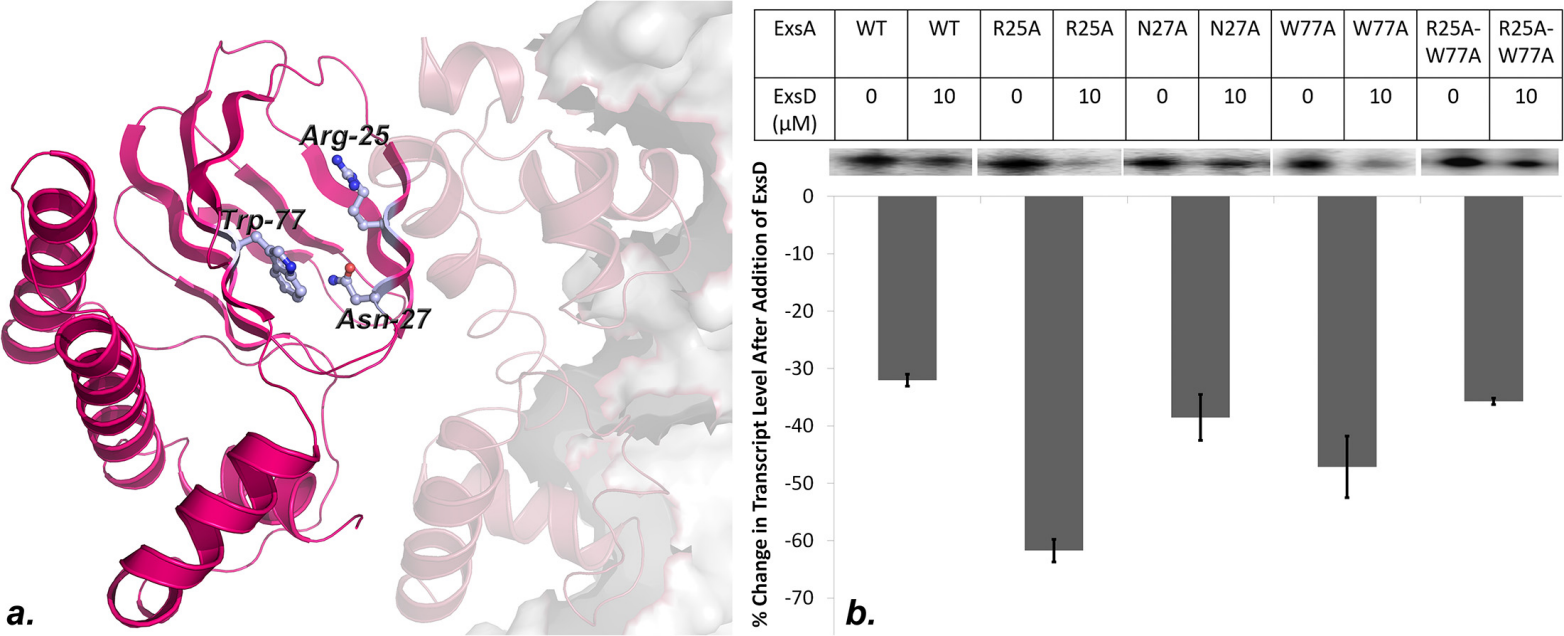
Impact of mutations in the conserved cavity of ExsA on ExsD binding. Three residues lining the cavity within the beta-sandwich structure of ExsA were mutated with alanine to determine if these residues are involved in ExsD binding. (A) Cartoon depiction of a full-length model of an ExsA-DNA complex. This model was generated by overlaying the structures of ExsA-NTD and a homology model of ExsA-CTD (based on the MarA-DNA crystal structure) onto the structure of ToxT. The mutated residues are depicted as ball-and-stick. (B) Results of *in vitro* transcription assays measuring the impact of the indicated mutations on ExsA-ExsD interactions. Plotted in the chart is the percent change in obtained transcript level when 10 μM ExsD is added to the reaction. A sample gel showing transcript bands is presented above the chart. Experiments were conducted in duplicate.

As pointed out above, ExsA belongs to a subgroup of AraC-type proteins, which are regulated through protein-protein contacts. Beyond knowing that the interactions are facilitated by the regulatory domain, we know very little about the location and properties of the interface. The structures of the ligand-bound forms of ToxT and AraC-NTD revealed binding pockets in virtually the same location within the core of the beta barrel structure [70, 75, 79]. Therefore, we sought to determine if the structurally equivalent region in ExsA also participates in ExsD binding. To test this hypothesis residues, R25, N27, and W77 were respectively replaced by alanines to generate three single substitution variants. In addition an ExsA-R25A-W77A double mutant was also expressed, purified, and tested. The selection of the to-be-modified residues was based on two criteria: together these amino acids line a small cavity at the entrance of the beta barrel core (Fig 3A). Moreover, R25 as well as W77 are not only structurally conserved in AraC but also interact with the arabinose ligand of that protein. All four variants of ExsA expressed stably and were shown to induce ExsA-dependent transcription *in vitro*. None of the four variants showed a statistically significant reduction in its sensitivity to ExsD (Fig 3B). In fact, the ExsA-R25A variant showed a statistically significant (P = 0.008) higher sensitivity to ExsD compared to wild-type. However, the effect was less pronounced with the ExsA-R25A-W77A double mutant (P = 0.09). Based on these results, we speculate that the mutations in the pocket have a slight effect on ExsA stability causing it to undergo the conformational changes induced by ExsD more readily.

## Discussion

**Fig 4:**
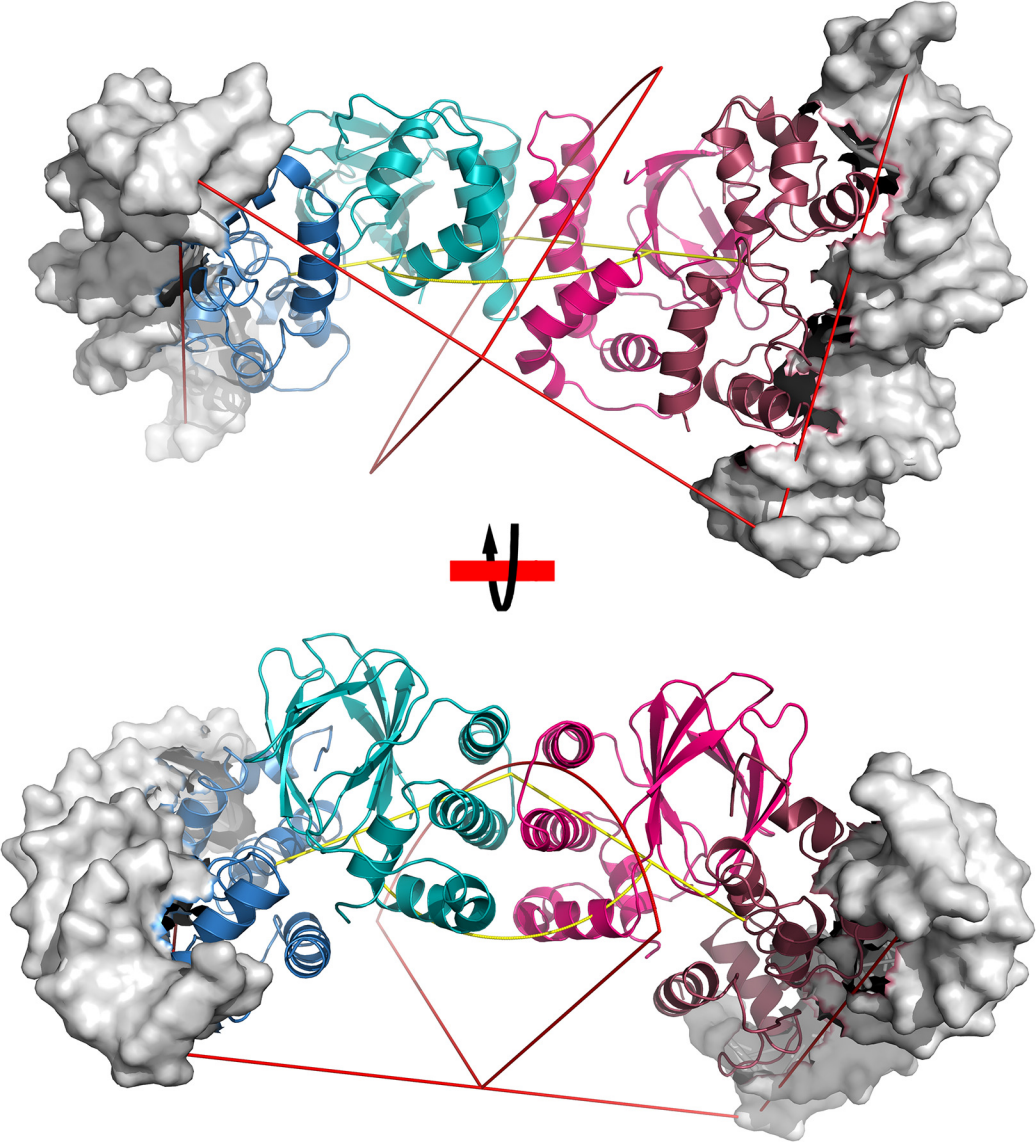
Cartoon model of a full-length dimeric ExsA-DNA complex. This model was generated by first overlaying the structure of the ExsA-NTD A/A’ dimer and a homology model of ExsA-CTD (based on the MarA-DNA crystal structure) onto the structure of ToxT. Subsequently, crystallographic two-fold axis was applied to create a model of the full-length protein with a dimer interface corresponding to A/A’ dimer observed in the crystal.

AraC-type proteins constitute a large family of transcription factors primarily found in bacteria and fungi [66]. Different family members have been shown to regulate stress response, metabolism, and virulence [66]. While prominent members of this group such as MarA are composed of only an AraC-type DNA binding domain and regulated at the transcriptional level [80], the canonical AraC-type transcription factor contains an additional amino-terminal regulatory domain [66]. Generally, binding of small molecule ligands within the regulatory domain modulates the function of the cognate transcription factor. The mechanism of regulation appears to differ among members of this protein family. In AraC, binding of arabinose triggers a rearrangement of an aminoterminal extension, which in turn permits the AraC dimer to bind to two adjacent binding sites on the promoter and activate transcription [81]. In ToxT, binding of a fatty acid molecule within a structurally conserved pocket causes a more compact interaction between N-and C domains to inhibit ToxT function [70]. ExsA belongs to an intriguing subgroup of AraC-like factors that is regulated not through interactions with a small-molecule ligand but by another protein[52]. In *P*.*aeruginosa* binding of the anti-activator ExsD interferes with both DNA-binding and homodimerization of ExsA [54, 55]. The latter process has recently been shown to be important for facilitating a conformational change in ExsA that permits consecutive binding of two ExsA molecules at adjacent sites on the promoter [65]. To date little is known about the structural basis of the ExsA-ExsD interactions or any of the other protein-protein complexes involving AraC-type transcriptional regulators. Despite a low level of sequence conservation the crystal structure of the ExsA-NTD closely resembles those of AraC and ToxT. In conjunction with previous work [65] our structural and experimental data suggest that the dimerization interface of ExsA is also conserved between ExsA and AraC. Yet, the ligand binding pocket used by AraC and ToxT appears to play no role in ExsD binding. The structure of the ToxT-fatty acid complex represents the only known structure of a full length AraC protein with the canonical domain architecture. Yet, this structure and biochemical studies of AraC highlight the mechanistic importance of the intramolecular interactions between NTD and CTD. For example, given the results of the present work and a recently published study, we now have an excellent idea about the location of the interface of the ExsA homodimer. Using the structures of full-length ToxT (pdb code: 3gbg) and the MarA-DNA complex (pdb code: 1bl0) [80] as templates we generated a model for a dimer of a full-length ExsA-DNA complex. In this model DNA binding domains are arranged at a 120° angle (Fig 4). Provided ToxT uses the same dimerization interface as AraC and ExsA this certainly explains why ToxT is inhibited by the fatty acid [70], however, it also raises the question what the “active” conformation of these proteins looks like. Is it a simple loosening of the domain interactions that is causing activation? If ExsA assumes similar conformations, does ExsD binding also encourage the formation of a rigid NTD-CTD interface in ExsA to inhibit transcription? At this point it has become quite clear that the regulatory mechanism of AraC is by no means representative for all AraC-type proteins. Therefore, perhaps contrary to general perception, much remains to be learned about this huge family of microbial transcription factors.

## Material and Methods

### Preparation of ExsD and ExsA variants

The genes coding for the ExsA variants genes were generated by site-directed mutagenesis using Quik-Change (Stratagene) kit and the manufacturer’s suggested protocol. The following primers were used:

*exsAW77A* 5’→3’: **F**: GGAAAGGACAGCCGAATACTCGCGATTCCATTATCTGCCC AGTTTCTACAAGGC; **R**: GCCTTGTAGAAACTGGGCAGATAATGGAATCGCGAGT ATTCGGCTGTCCTTTCC

*exsAR25A* 5’→3’: **F**: GTCATTGGAACATTCCAACTTTCGAATACGCGGTAAACAAG GAAGAGGGCGTATAT; **R**: ATATACGCCCTCTTCCTTGTTTACCGCGTATTCGAAA GTTGGAATGTTCCAATGAC

*exsAL117R* 5’→3’ **F**: CTGCCACGCCTCGGCTGGCCGGTG; **R**: CAACCGGCCAGCCG AGGCGTGGCAG

*exsAG124R* 5’→3’ **F**: GCCGGTTGCGTCAAGAGGTTGAAGGAATTGC; **R**: GCAATTC CTTCAACCTCTTGACGCAACCGGC

*exsAN27A* 5’→3’ **F**: GTCATTGGAACATTCCAACTTTCFAATACAGGGTAGCCAA GGAAGAGGGCGTATAT; **R**: ATATACGCCCTCTTCCTTGGCTACCCTGTATTCGA AAGTTGGAATGTTCCAATGAC

The *exsAW77A* gene served as template to create the *exsAR25A-W77A* double mutant using primers 5’→3’: **F:** GCCGGTTGCGTCAAGAGGTTGAAGGAATTGC; **R:** GCAATTCCT TCAACCTCTTGACGCAACCGGC

### Recombinant protein expression and purification

All ExsA variants and ExsD were overexpressed in *E*. *coli* from a vector constructed by Gateway recombinational cloning (Invitrogen, Carlsbad, CA, USA). A tobacco etch virus (TEV) protease recognition site and the appropriate att recombination sites (attB1 and attB2) were added to the *exsA* and *exsD* genes during PCR, and the amplicons were subsequently recombined into pDONR201 (Invitrogen). The nucleotide sequences of the ORFs were verified, then recombined into the destination vector pDEST-HisMBP [82] to create the expression vectors pFS-HMBPExsD and pFS-HMBPExsA. These vectors were designed to produce either ExsA or ExsD as a fusion to the C-terminus of an N- terminally His6-tagged *E*. *coli* maltose-binding protein (MBP).

Single colonies of *E*. *coli* BL21(DE3) CodonPlus RIL cells (Stratagene, La Jolla,CA, USA) containing either expression plasmid were used to inoculate 125 mL of Luria broth (LB) supplemented with 2 g/L dextrose, 100 μg/mL ampicillin, and 30 μg/mL chloramphenicol. The cultures were grown to saturation with shaking (225 rpm) overnight at 37°C and then diluted 66-fold into 6 L of fresh medium. pFS-HMBPExsA containing cultures were grown to an OD_600_ of 0.6–0.8 prior to induction with 1 mM isopropyl-β-D-thiogalacto-pyranoside (IPTG), while pFS-HMBPExsD containing cultures were induced at an OD600 of 0.6 using the same concentration of IPTG. ExsA expression was induced overnight at 18°C, whereas ExsD variants were expressed at 28°C for four hours. All cells were harvested by centrifugation at 5,000 x g for 15 minutes. For purification cell pastes were resuspended in 200 mL of 500 mM NaCl, 25 mM imidazole, 50 mM Tris-HCl (pH 7.4), 2 mM dithiothreitol (DTT) (buffer A), along with three tablets of Complete EDTA-free Protease Inhibitor Cocktail (Roche Applied Science, Indianapolis, IN, USA). Cells were lysed via sonication and centrifuged at 40,000 x g for 40 min. The supernatants were filtered through 0.45-μm polyethersulfone membranes and applied to a 30 mL Ni-NTA Superflow affinity column (Qiagen, Valencia, CA, USA) equilibrated with buffer A. For each run, the column was washed with five column volumes of buffer A, and proteins were eluted with a linear gradient from 25 to 250 mM imidazole (pH 7.4).

The His6-MBP-ExsD protein was digested with 5 mg His-tagged TEV (S219V) Protease [83] while being dialyzed overnight in 150 mM NaCl, 50 mM Tris-HCl (pH)7.4), 25 mM imidazole (pH 7.4), and 1 mM DTT. The sample was then passed through a second Ni-NTA column to remove both the His6-MBP tag and the tagged TEV protease, using the same buffers as employed for running the initial Ni-NTA column. The ExsD sample was obtained from the flow through. This sample was diluted with 50 mM Tris-HCl (pH 7.4) and 2 mM DTT in order to lower the NaCl concentration to 50 mM. The ExsD sample was loaded onto a HiTrap Q HP column (GE Healthcare, Waukesha, WI, USA) that had been equilibrated with 50 mM NaCl, 25 mM Tris-HCl (pH 7.4), and 2 mM DTT. Elution was achieved by applying a linear gradient of NaCl from 50 mM to 1 M. The final purification step involved loading the sample onto a HighLoad 26/60 Superdex 200 prep grade column (GE Healthcare) preequilibrated with a running buffer containing 150 mM NaCl, 25 mM Tris-HCl (pH 7.4), and 2 mM Tris (2-Carboxyethyl) phosphine (TCEP).

Purification of the ExsA protein followed a different protocol. Following the initial Ni-NTA affinity purification step, the His6-MBP-ExsA fusion protein was dialyzed against a buffer of 50 mM NaCl, 25 mM Tris-HCl (pH 7.4), and 2 mM DTT and loaded onto a HiTrap Q HP column (GE Healthcare) that had been equilibrated with the same buffer. The His6-MBP- ExsA fusion protein was eluted using a linear NaCl gradient from 0.05 M to 1 M. The sample was dialyzed against 2 L of 45 mM NaCl, 25 mM Tris-HCl (pH 7.15), and 2 mM DTT (buffer B) overnight. The sample was then loaded onto a HiTrap Heparin HP column (GE Healthcare) equilibrated in buffer B and eluted with a 0.05 M to 1 M gradient of NaCl. The NaCl concentration in the His-MBP-ExsA sample was adjusted to 0.5 M, and the fusion protein was digested with 3 mg of His_6_-TEV(S219V) protease at 4°C overnight. Next, ExsA was run through a second Ni-NTA Superflow affinity column, this time collecting ExsA in the flow through. Finally, the sample was run through a HighLoad 26/60 Superdex 200 prep grade column (GE Healthcare) using 500 mM NaCl, 25 mM Tris-HCl (pH 7.4), and 2 mM TCEP (ExsA storage buffer). The sample was concentrated to 1 mg/mL, flash-frozen using liquid nitrogen, and stored at -80°C. A sample SDS-gel of the purified proteins is provided with the supplemental materials (S3 Fig).

### Preparation and Crystallization of ExsA-NTD

ExsA full-length protein was digested by thermolysin (2 mg/mL) at 30°C for 1 hour, leaving only the N-terminal domain (verified by mass spectrometry). The product was applied onto a Superdex-26/60 gel-filtration column (GE Healthcare) (buffer: 500mM NaCl, 25mM Tris-HCl pH = 7.4, 2mM TCEP). The purified ExsA N-terminal domain protein was concentrated to 1.5mg/mL. Selenomethionine (SeMet) ExsA N-terminal domain protein was expressed in Escherichia coli BL21 (DE3) cells in minimal medium containing selenomethionine and was purified using the same protocol. As only properly folded protein can yield crystals, we wanted to show the purified ExsA-NTD protein is properly folded and functional. A titration of ExsA-NTD protein in the ExsA-dependent *in vitro* transcription assay is done to show that the ExsA-NTD protein can inhibit ExsA dependent transcription in a dose-dependent manner, which indicates that the purified ExsA-NTD protein competes for the dimerization site of full-length ExsA, resulting in a reduced transcription activity as the DNA binding motif is located in the C-terminal domain of ExsA. The result indicates the ExsA-NTD protein is properly folded and functional (S1 Fig). Crystallization of the ExsA-NTD was performed using the hanging-drop vapor-diffusion method at 25°C (S1 Fig). Crystals of ExsA-NTD were obtained using a reservoir solution containing 1.6M MgSO4, 0.1M MES pH 6.5 and 0.1M EGTA. The crystallization droplet consisted of 3μl protein solution (1.5 mg/mL ExsA N-terminal domain protein, 500mM NaCl, 25mM Tris-HCl pH = 7.4, 2mM TCEP) and 1μl reservoir solution. Rod-shaped crystals of ExsA-NTD appeared after several days. SeMet ExsA-NTD was crystallized under identical conditions. The crystals were flash-cooled in liquid nitrogen after soaking in a cryoprotection solution containing 90% reservoir solution and 10% (v/v) glycerol.

### X-ray diffraction data collection, structure determination and refinement

X-ray data were collected at beamline X29A (National Synchrotron Light Source, Brookhaven National Laboratory) using an ADSC Q315 CCD detector. The X-ray diffraction data were processed using the XDS program package. Initial phases for SeMet-ExsA-NTD were obtained by PHASER using the single anomalous dispersion [84]. Model building was performed using COOT [85], and the PHENIX program suite was used for structure solution and refinement [86]. During the refinement data were cut off at 2.5 Å using the correlation of the observed data set with the refined model, CC1/2 as defined by Karplus and Diederichs [87] as selection criterion. Data collection and refinement statistics are summarized in Table 1. The schematic representation of the non-bonding contacts in the A/A’ and B/B” dimers in S2 Fig was generated using PDBsum [88]. The refined model was deposited in the protein data bank under the accession code 4ZUA.

**Table 1:** Diffraction data and crystal structure refinement statistics.

Wavelength (Å)	1.075
Resolution range (Å)	56.51–2.5 (2.589–2.5)^a^
Space group	P 4_3_2_1_2
Unit cell (Å)	a = b = 69.9, c = 191.8
Total reflections	1,261,113 (118,691)^a^
Unique reflections	17,161 (1,581)^a^
Multiplicity	73.4 (75.1)^a^
Completeness (%)	99.36 (94.05)^a^
Mean I/sigma (I)	91.07 (4.53)^a^
Wilson B-factor (Å^2^)	57.80
^b^Rmerge	0.86 (4.107)^a^
^c^CC_1/2_	0.96 (0.495)^a^
^d^CC*	0.99 (0.814)^a^
^e^R_work_	0.239 (0.393)^a^
^f^R_free_	0.268 (0.403)^a^
Number of non-hydrogen atoms	2,401
Macromolecules	2,390
Water	11
Protein residues	306
RMS (bonds) (Å)	0.01
RMS (angles) (°)	1.38
Ramachandran favored (%)	93
Ramachandran outliers (%)	0.67
Clash score	8.66
Average B-factor (Å^2^)	77.7
Macromolecules (Å^2^)	77.8
Solvent (Å^2^)	61.4

^a^The values in parentheses relate to the highest resolution shell from 2.589–2.5Å.

^b^R_merge_ = Σ|I|- 〈I〉/ΣI, where I is the observed intensity, and I is the average intensity obtained from multiple observations of symmetry-related reflections after rejections.

^c^CC_1/2_ = Pearson correlation coefficient between random half-datasets

^d^CC* = [(2CC1/2)/(1+CC1/2)]^0.5^

^e^R_work_ = Σ||F_o_|—|F_c_||/Σ|F_o_|, where F_o_ and F_c_ are the observed and calculated structure factors, respectively.

^f^R_free_ defined in Ref. [72].

#### ExsA-dependent in vitro transcription assays

A previously published report indicated that *E*.*coli* RNA polymerase is can substitute for its *P*.*aeruginosa* counterpart in ExsA-dependent *in vitro* transcription assays [59]. Preliminary tests confirmed this observation. Therefore, in the interest of time all ExsA variants were analyzed using commercially available *E*.*coli* RNAP holoenzyme (Epicentre Biotech).

The linear DNA template used in each assay encompassed positions -207 to 94 of the P*exsD* promoter, relative to the transcription start site; and from this template, RNA polymerase synthesizes an 82 base mRNA transcript. The template was produced by PCR using forward primer 5´-CATCAGTTGCTGCTCAACAGCG-3´ and reverse primer 5´-CACCGCTTCTCGGGAGTACTGC-3´. The PCR product was run on a 2% agarose gel and purified using the Wizard SV Gel and PCR Clean-up System (Promega, Madison, WI, USA). Each 30 μL transcription assay reaction contained 4.4 fmole of promoter template, 50.4 μM bovine serum albumin (to eliminate non-specific protein-protein interactions), 10 U purified RNA polymerase holoenzyme, 1 U RiboGuard RNase Inhibitor (Epicentre Biotechnologies), 15 ng/μL poly(deoxyinosinic- deoxycytidylic) acid (to prevent non-specific transcription initiation), 133 mM NaCl, 32 mM Tris-HCl (pH 7.4), 10 mM MgCl2, 25 μM EDTA, 0.9 mM TCEP, 0.2 mM DTT, and 15.5% glycerol. The time-course experiments contained 64 nM ExsA and either no ExsD or 50 μM ExsD. Samples were mixed and allowed to equilibrate at room temperature for five min. Samples were then pre-incubated for 10 min at 37°C. Next, 3 μL NTPs (stock concentrations of 200 μM ATP, CTP, GTP and 40 μM UTP) mixed with 0.2 μL (0.2 μCi) of 3.3 mM P32-alpha UTP was added to each sample to start the reaction,and samples were incubated at either 30 or 37°C, depending on the experiment. After the reactions were stopped by adding 12 μL 1X stop solution (3M ammonium acetate, 50 mM EDTA, 0.11 mg/mL glycogen), 170 μL 100% cold ethanol was added, and the samples were incubated at -20°C for one hr. Following centrifugation at 12,000 x g for 15 min, the supernatant was discarded and pellets were resuspended in 12 μL 1X TBE (Tris/Borate/EDTA)-urea sample buffer and heated at 70°C for five min. After a brief centrifugation, the samples were loaded onto a 10% TBE-urea gel and run at 200 mV for 60 min. Gels were exposed to a storage phosphor screen (GE Healthcare) for 16 hrs. The phosphor screen was scanned using a Typhoon Trio Variable Mode Imager (GE Healthcare), and gel bands were quantified using Image Quant TL v2005 (Amersham Biosciences, Piscataway, NJ, USA). Each experiment was performed in duplicate.

## Supporting Information

S1 Fig (A) SDS-PAGE analysis of the purification of ExsA-NTD after digestion of ExsA with thermolysin via gel filtration chromatography. (B) Titration of ExsA N-ternimal domain in ExsA dependent transcription. The *in vitro* transcription assay was performed as described under Materials and Methods. In addition to the usually added 64 nM of ExsA WT samples contained the indicated concentration of the ExsA-NTD protein. (C) Crystals and sample X-ray diffraction image of ExsA-NTD. (TIF)

S2 Fig Schematic representation of the non-bonding contacts in the A/A’ and B/B” dimers of ExsA-NTD. The width of the dashed lines is proportional to the number of contacts between the residues. This schematic was generated using PDBsum [88].
(TIF)

S3 Fig SDS-PAGE slices of the purified protein samples used in the various experiments. (TIF)
