# Supplementary figures and images for: Structural Analysis of the Regulatory Domain of ExsA, a Key Transcriptional Regulator of the Type Three Secretion System in Pseudomonas aeruginosa
Source: PLoS One. 2015 Aug 28;10(8):e0136533. doi: 10.1371/journal.pone.0136533 (PMC4552939; doi:10.1371/journal.pone.0136533)

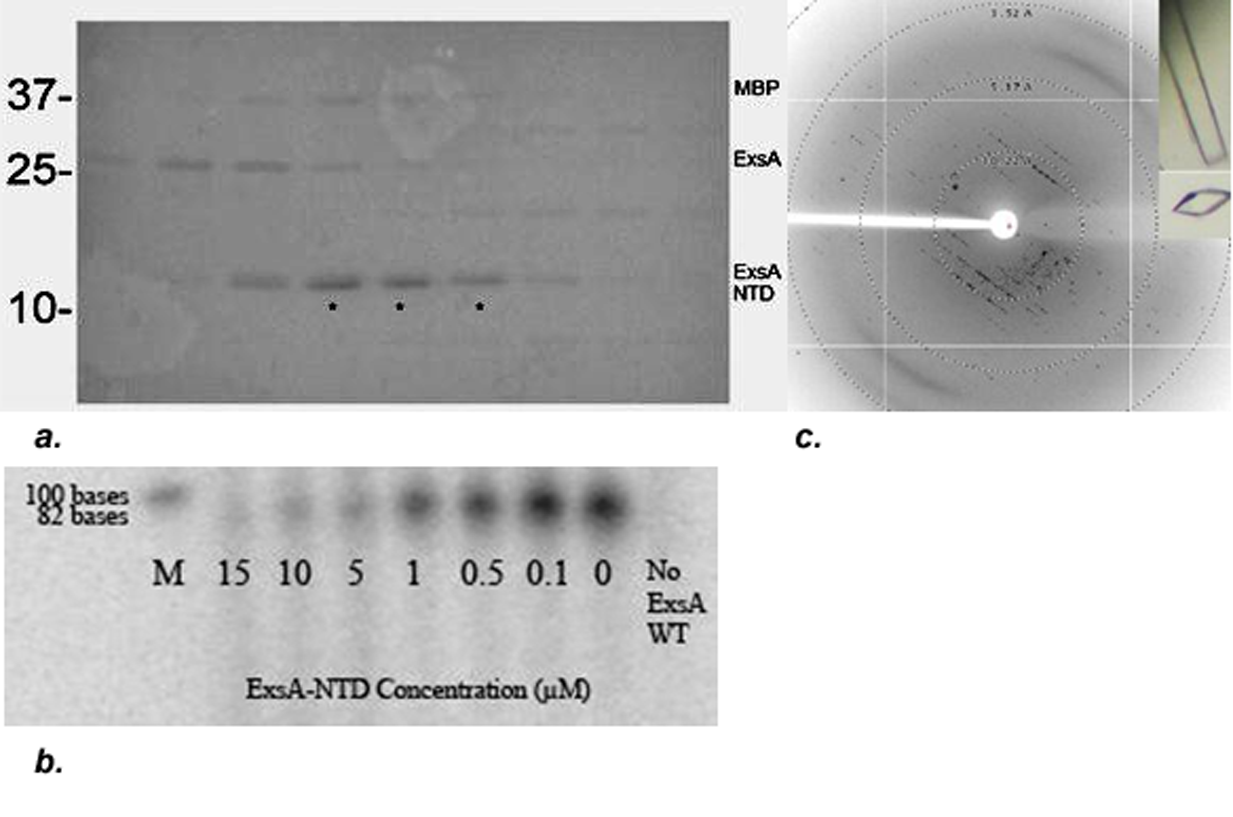

Supplement: S1 Fig — (TIF) [file pone.0136533.s001.tif]

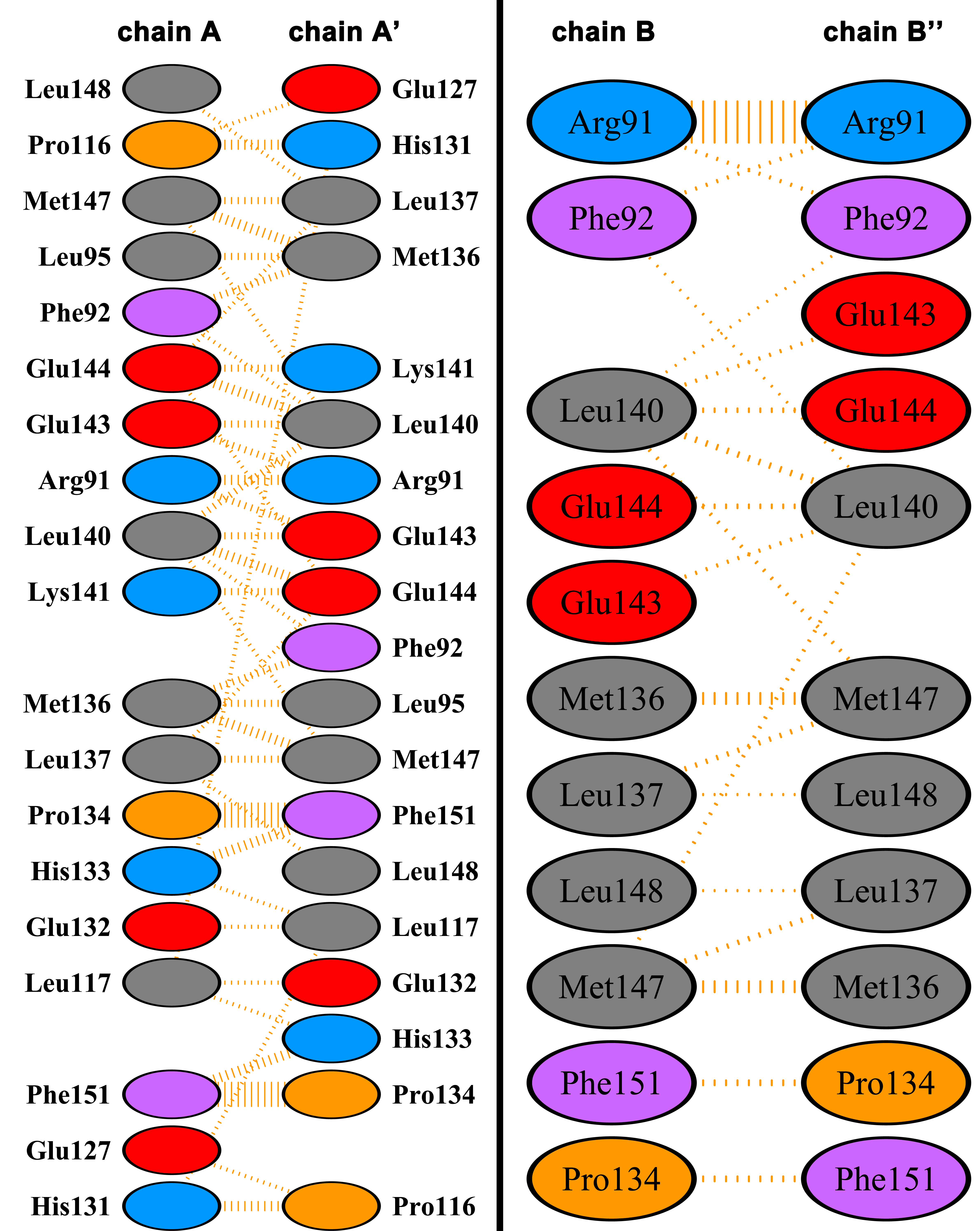

Supplement: S2 Fig — The width of the dashed lines is proportional to the number of contacts between the residues. This schematic was generated using PDBsum [88]. (TIF) [file pone.0136533.s002.tif]

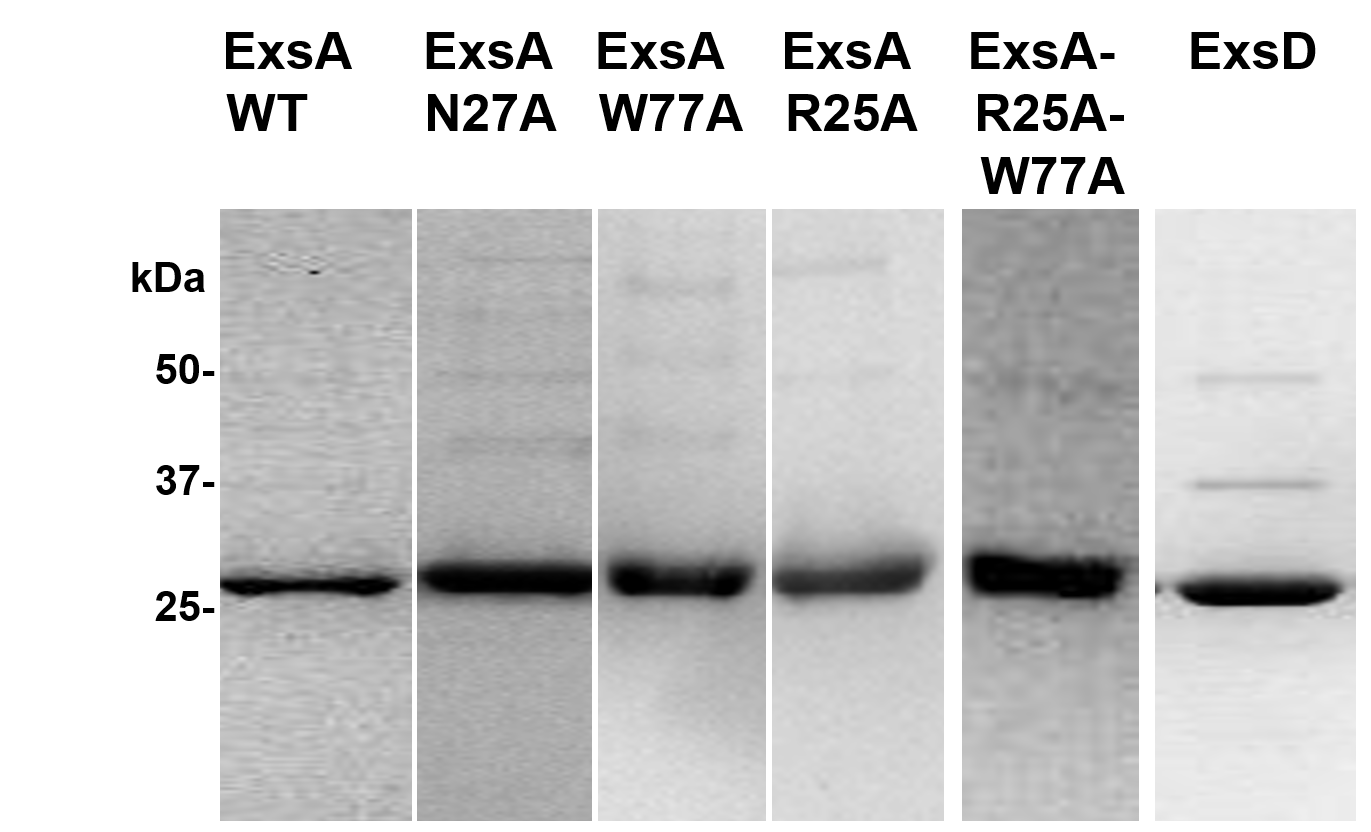

Supplement: S3 Fig — (TIF) [file pone.0136533.s003.tif]
